# Gene conversion and duplication contribute to genetic variation in an outbreak of Mycobacterium tuberculosis

**DOI:** 10.1099/mgen.0.001396

**Published:** 2025-05-01

**Authors:** Christoph Stritt, Michelle Reitsma, Ana Maria Garcia Marin, Galo Goig, Anna Dötsch, Sonia Borrell, Christian Beisel, Iñaki Comas, Daniela Brites, Sebastien Gagneux

**Affiliations:** 1 Swiss Tropical and Public Health Institute, Allschwil, Switzerland; 2 University of Basel, Basel, Switzerland; 3 Biomedicine Institute of Valencia, Spanish Research Council (IBV-CSIC), Valencia, Spain; 4 Department of Biosystems Science and Engineering, ETH Zurich, Basel, Switzerland; 5 Spanish Network for Research on Epidemiology and Public Health (CIBERESP), Carlos III Health Institute, Madrid, Spain

**Keywords:** long-read sequencing, repeats, recombination, pangenome graph, PE/PPE genes, phylogenetic resolution

## Abstract

Repeats are the most diverse and dynamic but also the least well-understood component of microbial genomes. For all we know, repeat-associated mutations such as duplications, deletions, inversions and gene conversion might be as common as point mutations, but because of short-read myopia and methodological bias, they have received much less attention. Long-read DNA sequencing opens the perspective of resolving repeats and systematically investigating the mutations they induce. For this study, we assembled the genomes of 16 closely related strains of the bacterial pathogen *Mycobacterium tuberculosis* from Pacific Biosciences HiFi reads, with the aim of characterizing the full spectrum of DNA polymorphisms. We found that complete and accurate genomes can be assembled from HiFi reads, with read size being the main limitation in the presence of duplications. By combining a reference-free pangenome graph with extensive repeat annotation, we identified 110 variants, 58 of which could be assigned to repeat-associated mutational mechanisms such as strand slippage and homologous recombination. Whilst recombination events were less frequent than point mutations, they affected large regions and introduced multiple variants at once, as shown by three gene conversion events and a duplication of 7.3 kb that involved *ppe18* and *ppe57*, two genes possibly involved in immune subversion. The vast majority of variants were present in single isolates, such that phylogenetic resolution was only marginally increased when estimating a tree from complete genomes. Our study shows that the contribution of repeat-associated mechanisms of mutation can be similar to that of point mutations at the microevolutionary scale of an outbreak. A large reservoir of unstudied genetic variation in this ‘monomorphic’ bacterial pathogen awaits investigation.

**Impact Statement**

Some of the most ancient and virulent pathogenic bacteria – including the agents of anthrax, typhoid, plague, leprosy and tuberculosis – are characterized by low levels of genetic diversity. Whilst this is partly due to the extreme clonality and absence of horizontal gene transfer in these organisms, it also reflects technological limitations: previous approaches to genotyping and sequencing could only consider a subset of variant types or genomic regions. In this study, we assembled essentially error-free genomes from long Pacific Biosciences HiFi reads and used a reference-free pangenome graph to characterize the full spectrum of genetic variation in the context of an outbreak of the bacterial pathogen *Mycobacterium tuberculosis*. We show that regions and variant types typically ignored by short-read sequencing studies contribute more than half of the genetic variation observed in 16 outbreak strains and increase our ability to distinguish one strain from another in this low-diversity bacterial pathogen. Whilst our small sample does not lend itself to generalizations, it provides a detailed analysis of how homologous recombination between repeats resulted in gene conversion and duplication – mutational processes whose impact in the evolution of the MTBC we are only beginning to understand. Even in our small sample, these processes affected genes that seem to play a role in immune subversion and are part of vaccine candidates.

## Data Summary

For this study, 17 strains of the *Mycobacterium tuberculosis* complex (lineage 4) were sequenced with a Pacific Biosciences Sequel II machine in circular consensus sequencing (or HiFi) mode. The raw consensus reads and the assembled genomes were deposited on the European Nucleotide Archive (PRJEB73759). Illumina short-read sequencing data for the same strains have been published previously (PRJEB5925). The genome assembly pipeline is available as a Snakemake workflow on http://git.scicore.unibas.ch/TBRU/PacbioSnake, and detailed commands and scripts used are provided in a Jupyter notebook on https://github.com/cstritt/repeats_mtbc.

## Introduction

DNA repeats are the most diverse and dynamic components of any genome, not counting viruses. They comprise a veritable zoo of elements of different origins and complexities, ranging from short tandem repeats (TRs) and subtle palindromes to autonomously replicating transposable elements and members of multigene families (for a comprehensive review, see [1]). Whilst the molecular evolution of repeats is highly variable, they share the property of providing a substrate for homologous or illegitimate recombination. This makes them the principal cause of genome instability and DNA polymorphisms in the form of duplications, deletions, inversions and non-reciprocal transfers between homologues through gene conversion [2].

Whole-genome sequencing has shown that repeat-associated variation is ubiquitous. This insight stems from dedicated studies (e.g. [3,4]), but maybe even more from frustrated attempts to assemble genomes or identify variants using reads that are too short to span repeats, which results in fragmented assemblies and ambiguous mappings. Along with short-read myopia comes a methodological bias towards point mutations, which are simpler to model and underlie downstream analyses such as phylogenetic inference and selection scans. For most organisms, little is therefore known about the types, rates and phenotypic effects of repeat-associated mutations. Recently, a systematic investigation of repeats has come within reach thanks to long-read sequencing and analytical tools such as pangenome graphs [5] and hierarchical alignment [6]. For the streamlined genomes of prokaryotes, base-perfect assemblies can be generated [7], and pangenome graphs can be used to obtain a concise representation of all variant types [8].

One area in which a full characterization of genetic diversity would be particularly useful is the study of bacterial pathogens. Some of the most ancient and virulent of them – including the agents of anthrax, typhoid, plague, leprosy and tuberculosis – have been designated ‘monomorphic’ because of their low levels of genetic diversity [9]. Lack of horizontal gene transfer (HGT) and clonal evolution contribute to the phenomenon of ‘monomorphy’, although the population genetics of extreme clonality remains poorly understood [10]. In the absence of HGT, intrachromosomal recombination between repeats might be an important mutational mechanism in these bacterial pathogens. As in other organisms, however, the study of repeats and structural variants has been neglected since the advent of short-read sequencing, and we remain largely ignorant about their contribution to genetic and phenotypic variation.

In this study, we used Pacific Biosciences (PacBio) HiFi sequencing to characterize the full spectrum of DNA polymorphisms in 16 strains of *Mycobacterium tuberculosis*, the agent of tuberculosis (TB), which, together with other closely related lineages, forms the *M. tuberculosis* complex (MTBC [11]). We focused on a transmission cluster in the city of Bern, Switzerland, previously characterized through restriction fragment length polymorphisms [12] and Illumina sequencing [13,14]. The cluster reflects mainly transmission amongst homeless and substance abusers in the 1990s, with spillovers to the general population and reactivated TB diagnosed up to 2012 [13]. This is not the typical manifestation of TB, which, today, mainly affects low-income countries and is, following the receding of the COVID-19 pandemic, again the most deadly infectious disease in the world [15]. How *M. tuberculosis* manages to be so successful with so little genetic diversity remains puzzling. Part of the answer may lie in the genomic ‘dark matter’, the repetitive sequences in the genomes of these bacteria.

Here, we unlock the repeatome of the MTBC by addressing a simple question: what types of variants are there? More specifically, we (1) evaluate the accuracy of assemblies constructed from PacBio HiFi reads, (2) describe the different types of DNA polymorphisms and their underlying mutational mechanisms and (3) investigate how considering complete genomes rather than only SNPs in non-repetitive regions affects genetic distances and phylogenetic resolution.

## Methods

### Samples and genome sequencing

Strains for PacBio sequencing were selected to represent the ‘Bernese outbreak’, a small transmission cluster belonging to lineage 4 and including 68 patients in the city of Bern (Switzerland), sampled between 1988 and 2011 (Fig. S1, Table S1, available in the online Supplementary Material [12,13]). We first estimated a phylogenetic tree using the previously published short reads (NCBI BioProject PRJEB5925). An SNP alignment was created as previously described [16], ignoring variants in known resistance genes and repetitive regions. The resulting alignment of 142 variable positions was used to estimate a phylogeny with raxml-ng v.1.2.1 [17], using the generalized time reversible substitution model and 100 bootstrap replicates. Branch lengths were rescaled to account for non-variable positions.

Sixteen strains from across the outbreak tree were selected for sequencing. For two patients (P022 and P028), different isolates than the ones used by Stucki *et al*. were accidentally selected. We still used them and unambiguously labelled the assemblies with the patient number and the isolate name separated by a dash. For DNA extraction, strains were grown for 2–3 weeks on 7H11 plates inoculated from 7H9 liquid wake-up cultures. DNA was extracted from the plates using a method based on cetrimonium bromide [18] that includes RNAse treatment and a purification step using magnetic beads. SMRTbell libraries were prepared at the Genomics Core Facility jointly run by ETH Zurich and the University of Basel and sent for sequencing on a PacBio Sequel II System at the Lausanne Genomic Technologies Facility.

### Genome assembly and variant calling

Read quality statistics were obtained with LongQC [19]. We used Flye v. 2.8.1 [20] to assemble genomes from circular consensus sequences (CCS) and reoriented the assemblies to start with *dnaA* using Circlator v. 1.5.5 [21]. As *M. tuberculosis* is not known to contain plasmids, assembly is expected to result in a single circular chromosome. A pangenome graph representation of the assembled single-contig genomes was built using PanGenome Graph Builder (PGGB) v. 077830d [5], which includes the *vg* and *odgi* toolsets for variant calling and graph manipulation. The minimum pairwise identity between seeds (*-p*) and the seed length (*-s*) are important parameters of PGGB that affect the structure of the pangenome graph, particularly when considering diverse genomes [8]. Since the genomes analysed in our study are highly similar, we set *-p* to 99, whilst *-s* was set to 5 k according to the comparatively short length of repeats expected in the assemblies. An arbitrary strain, P034-N1426, was used as a positional reference for outputting variants with *vg deconstruct* and for the annotation of repeats (described below).

To classify structural variants as insertions or deletions, we estimated the ancestral states of the variants by comparing them to a closely related outgroup strain. The Bernese strains belong to sublineage 4.4.1.1 according to the published short-read data and the SNP barcode of Coll *et al*. [22]. From our in-house database, we selected N1015 (ERR233382), a strain from Nepal, as an outgroup because it belongs to sublineage 4.4.1.2 and was physically available for cultivation and DNA extraction. The genome of this strain was also sequenced and assembled, as described above (Table S1). To infer the ancestral states of variants within the outbreak sample, the corresponding allele in N1015 was assumed to represent the ancestral state.

### Assembly validation and curation

To evaluate the accuracy of our assemblies, we looked for inconsistencies between reads and assemblies by aligning the long reads back against the assemblies and calling variants. If the assembly is accurate, no variants should be found, whilst variants identified this way indicate errors during assembly, circularization or the presence of true genetic variation in the culture. We used minimap2 2.24-r1122 [23] to align the reads and called variants with freebayes v. 1.3.4 [24], setting ploidy to 1. Read-assembly inconsistencies were scrutinized with the Integrative Genomics Viewer [25], and those unequivocally identified as assembly errors were corrected using the *pysam* library in Python. The corrected assemblies were validated through a second round of variant calling.

### Gene and repeat annotation

Genes were annotated *de novo* in all assemblies with bakta v.1.8.2 [26]. To compare gene models, we also lifted over the H37Rv reference annotation (ASM19595v2) to P034-N1426 with liftoff v1.6.3 [27]. To further characterize the repeat context of the variants, we annotated different types of repeats in the reference strain P034-N1426: insertion sequences (IS) with ISEScan v.1.7.2.3 [28], short sequence repeats (SSRs) (<=9 bp) with kmer-ssr v. 0.8 [29], tandem repeats (TRs, >9 bp) with SPADE v. 1.0.0 [30] and homopolymers of at least 5 bp using our own script. To investigate these repeats in other assemblies, annotations were lifted over using *odgi position* on the pangenome graph. The variant positions resulting from *vg deconstruct* and the different annotations were intersected with bedtools v2.30.0 [31].

As a second approach to characterize the repeat landscape, we identified interspersed repeats of at least 50 bp and 90% identity across the genome. Sequence homology is the substrate for homologous recombination and thus informative about where in the genome we might expect recombination-associated mutations. The thresholds are somewhat arbitrary as the minimal efficient processing segment for homologous recombination is not known in the MTBC; this is addressed in the discussion. We used *nucmer* v. 3.1 (–maxmatch –nosimplify) and *show-coords* from the MUMmer tool v.3.23 [32] and removed self- and overlapping hits as well as hits overlapping with annotated TRs. The locations of homology segments were intersected with the *de novo* gene and IS annotations using bedtools v2.30.0 [31] in order to identify features that could potentially recombine.

### Identification of gene conversion tracts

Gene conversion is the non-reciprocal transfer of a stretch of DNA from a source gene to a homologous target gene. The characteristic signature of such an event is a mosaic gene structure with a dense cluster of variants in the converted region of the target gene. To identify gene conversion events, we first traversed the VCF file resulting from *vg deconstruct* and identified clusters of variants less than 500 bp apart and occurring in the same strains. Given the low overall diversity of our sample, nearby variants in the same strain stand out clearly, and clustering is robust to different distance thresholds. We then extracted the regions containing the variants – the suspected conversion tracts – from the respective genomes using *odgi position* and samtools v.1.18 [33] and used blastn v. 2.12.0 [34] to align the sequences against (a) the genome in which the tract was detected and (b) a genome lacking evidence for gene conversion in the region under consideration. In the case of gene conversion, we expect two matches in (a), in the source and the target gene, and a single match in (b), i.e. only in the source gene. Visualization of the gene conversion and duplication events was done using dotter [35] and the R package *gviz* [36]. To investigate whether gene conversion affected epitope regions, epitope sequences were downloaded from the Immune Epitope Database (iedb.org, accessed 24.1.2024). Miniprot v. 0.13 [37] was used to infer the location of the epitope peptides within the nucleotide sequences of the genes.

### Comparison with Illumina data

In order to understand to what degree fully resolved repeats increase genetic resolution and our ability to distinguish closely related strains, we compared genetic distances and phylogenetic trees based on published short-read data with distances and trees based on our pangenome graph. For the former, we used the SNP alignment constructed from the published short reads, as described above, which includes variants in non-repetitive regions (‘Illumina SNPs’). From the pangenome graph, two alignments were extracted after calling variants relative to the arbitrary reference P034-N1426: firstly, an alignment of SNPs in the core genome, which was obtained by considering only SNPs in nodes shared by all 16 strains (‘PacBio SNPs’), and secondly, an ‘alignment’ of all variant types, where we artificially coded indels and structural variants as nucleotide bases (‘PacBio all’). The R package ape v.5.8 [38] was used to calculate pairwise Hamming distances [dist.dna(model=’N’)] for the three alignments and to estimate phylogenetic trees using neighbour-joining and 1,000 bootstrap replicates. Trees were visualized with ggtree v.3.12.0 [39].

## Results

### Complete and accurate assemblies from CCS reads

Sixteen strains from the Bernese outbreak, isolated between 1988 and 2005, were selected for sequencing on a PacBio Sequel II in CCS mode (Fig. S1, Table S1). Using the Flye assembly algorithm, all but one genome (P001-N1377) could be assembled into single circular chromosomes, despite considerable variation in read lengths and sequencing depths (Table 1). Indeed, a median depth of 16 was sufficient to obtain a closed genome with only one single ambiguous position (see below). To test whether assemblies were not only complete but also accurate, we aligned the long reads back against the respective assemblies and called variants to discover inconsistencies between the two. Five sites in four assemblies were identified where the reads contradict the assembly, whilst 12 assemblies are free of inconsistencies and appear to be correct to the base. A closer inspection of the inconsistencies suggests that they are due to different causes (Fig. S2). In P003-N1374, the assembly with the lowest mean coverage (16×), a total of five reads disagree on a sequence of five versus four cytosines. A second case, in P028-N1362, seems to reflect genuine heterogeneity as it might arise during culture or from a heterogeneous inoculum, with 28 reads supporting an adenine versus 28 reads supporting a guanine. A third inconsistency arises from a duplication in P001-N1377 (discussed below). This sequence was not resolved by Flye, but in the subsequent circularization step, where 12 bp evident in the reads went missing. Finally, two nearby single-base insertions in P034-N1426 suggested by the assembly but not the reads are due to misassembly: one single read shows the presence of the two additional bases, whilst 79 contradict it. The last two inconsistencies, which are clear assembly errors, were corrected: 12 bp were added to the duplication in P001-N1377, and two bases were deleted from P034-N1426.

**Table 1.** Read and assembly statistics. Read information was obtained from the LongQC output, and assembly information from the Flye summary files

		Reads				Assemblies		
**Isolate**	**Year**	**Nr reads**	**N50**	**Longest**	Q7*	**Length**	**Coverage†**	**Circular**
P007-N1108	2005	44,212	5344	21,567	99.79	4,405,381	47	Y
P028-N1362	na	43,856	5961	27,471	99.79	4,405,379	56	Y
P003-N1374	1991	11,692	6726	24,804	99.75	4,405,378	16	Y
P001-N1377	1991	26,848	6839	27,922	99.74	4,412,646	38	N
P008-N1380	1991	24,578	7534	27,155	99.70	4,405,372	38	Y
P010-N1385	1991	53,811	5391	21,423	99.79	4,405,379	58	Y
P020-N1386	1992	29,983	7884	27,587	99.69	4,405,380	48	Y
P059-N1392	2002	76,540	4781	15,395	99.85	4,405,268	70	Y
P073-N1394	1998	38,388	7422	27,900	99.72	4,405,232	59	Y
P074-N1402	1993	31,862	7594	29,206	99.70	4,405,232	50	Y
P066-N1411	2003	26,719	7075	28,501	99.72	4,405,380	39	Y
P034-N1426	1995	66,552	6740	27,519	99.77	4,405,492	90	Y
P052-N1429	1995	26,244	7941	27,868	99.68	4,405,378	43	Y
P042-N1430	1996	18,475	7657	26,514	99.71	4,405,378	29	Y
P022-N1431	na	40,133	7474	27,860	99.73	4,405,379	62	Y
P006-N1591	1988	42,575	7713	27,040	99.72	4,405,380	68	Y
N1015‡	na	28,637	6827	27,022	99.75	4,406,639	41	Y

*Percentage of bases with quality >7. †Median. ‡Nepalese outgroup strain belonging to lineage 4.4.1.2.

### The repeat landscape of *M. tuberculosis*

**Fig. 1.**
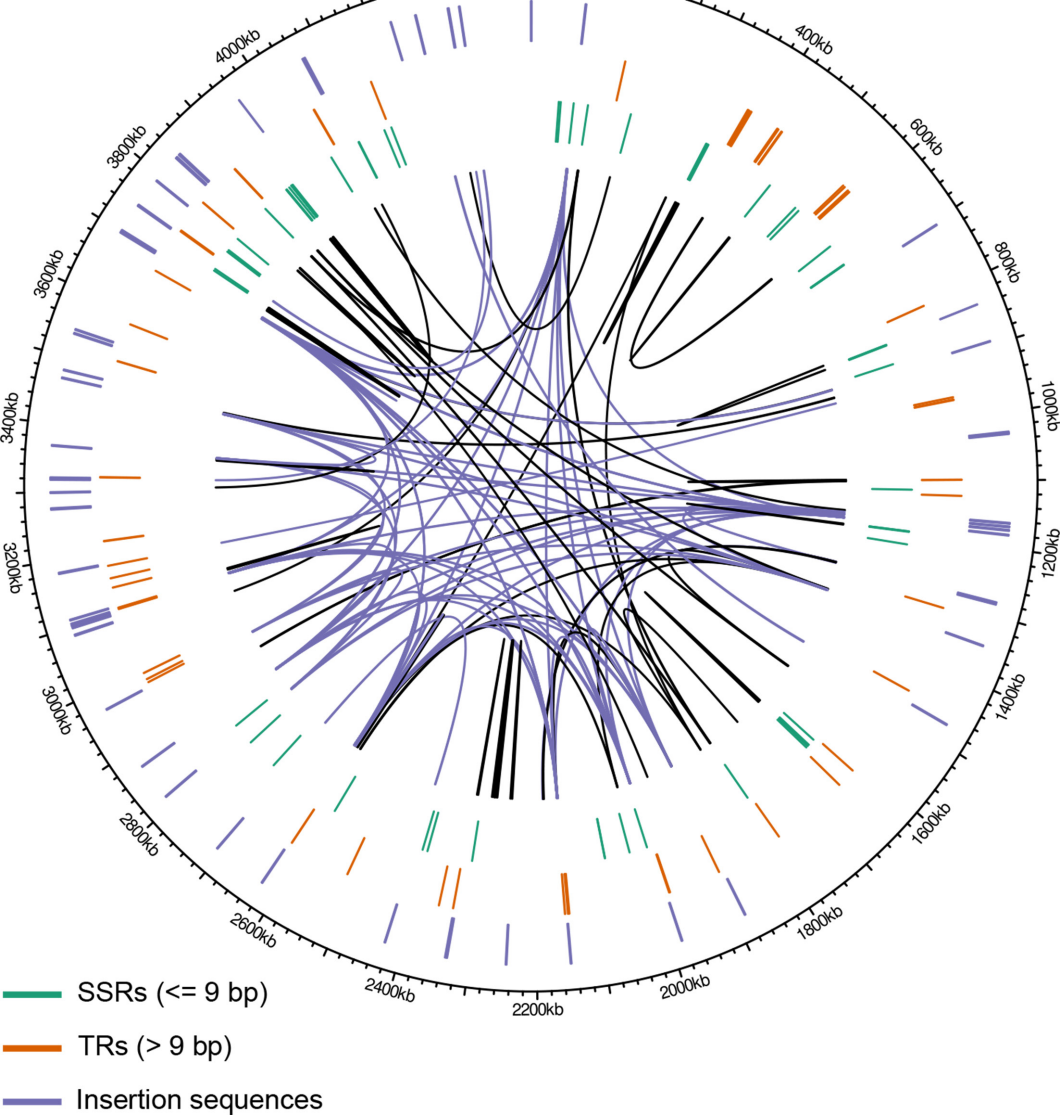
Repeat landscape in the reference isolate P034-N1426. Annotated SSRs (<=9 bp, *n*=67), TRs (>9 bp, *n*=47) and insertion sequences (*n*=57) are shown in the three outer tracks. Homopolymers are not shown because of their large number (*n*=6,770). The links inside the circle show interspersed repeats of at least 90% identity and 50 bp length (*n*=136 pairs). Links that connect copies of insertion sequences are shown in purple, others in black. The total repeat content of the genome – including SSRs, TRs, homopolymers and interspersed repeats – is 3.2% or 142,024 bp.

Considering the key role of repeats in causing structural variation and gene conversion, we first sought to understand the repeat landscape in the studied genomes. Different types of repeats were annotated in P034-N1426, the genome used as a positional reference for variant calling (Fig. 1): homopolymers of at least 5 bp, SSRs (direct repeats of 3–9 bp), TRs (direct repeats>9 bp) and insertion sequences. Homopolymers were the most abundant type with 6,770 occurrences. Sixty-seven SSRs were identified, the large majority of them triplets of six or nine that do not shift reading frames (Fig. S3A). TRs were found at 47 locations, with quite some variation in repeat periodicity and length (Fig. S3A). Finally, 57 insertion sequences were identified, including 12 copies of IS6110 (IS3 family) and 9 copies of IS1081 (IS256 family), two IS families in the MTBC that are known to vary in copy number.

As a second approach to characterize the repeat landscape, we identified interspersed repeats of at least 50 bp and 90% identity across the genome. One hundred thirty-six pairs of interspersed repeats were identified, making up a total of 93,685 bp or 2.1% of the 4.4 Mb genome (Fig. 1). To better understand which genetic elements share sequence homology, we intersected the interspersed repeats with the gene and IS annotations. The repeat landscape is dominated by 45 and 15 pairs of highly similar IS6110 and IS1081 copies, respectively (Fig. 1). Members of the ESX, PE and PPE gene families constitute a second prominent feature: nine ESX genes (*esxI*, *esxJ*, *esxK*, *esxL*, *esxN*, *esxO*, *esxP*, *esxV* and *esxW*), eight PPE genes (*ppe18*, *ppe19*, *ppe34*, *ppe38*, *ppe46*, *ppe57*, *ppe59* and *ppe60*) and seven PE_PGRS genes (*pe_pgrs17*, *pe_pgrs18*, *pe_pgrs19*, *pe_pgrs20*, *pe_pgrs27*, *pe_pgrs28* and *pe_pgrs45*) are part of interspersed repeats (Fig. S3B). Most of the remaining sequence homology is found between genes of unknown function. Combining our repeat annotations (SSRs, TRs, homopolymers and interspersed repeats), we found that 3.2%, or 142,024 bp, of the P034-N1426 genome is repetitive (Fig. 1).

### Types, frequencies and genomic context of the 110 identified variants

**Fig. 2.**
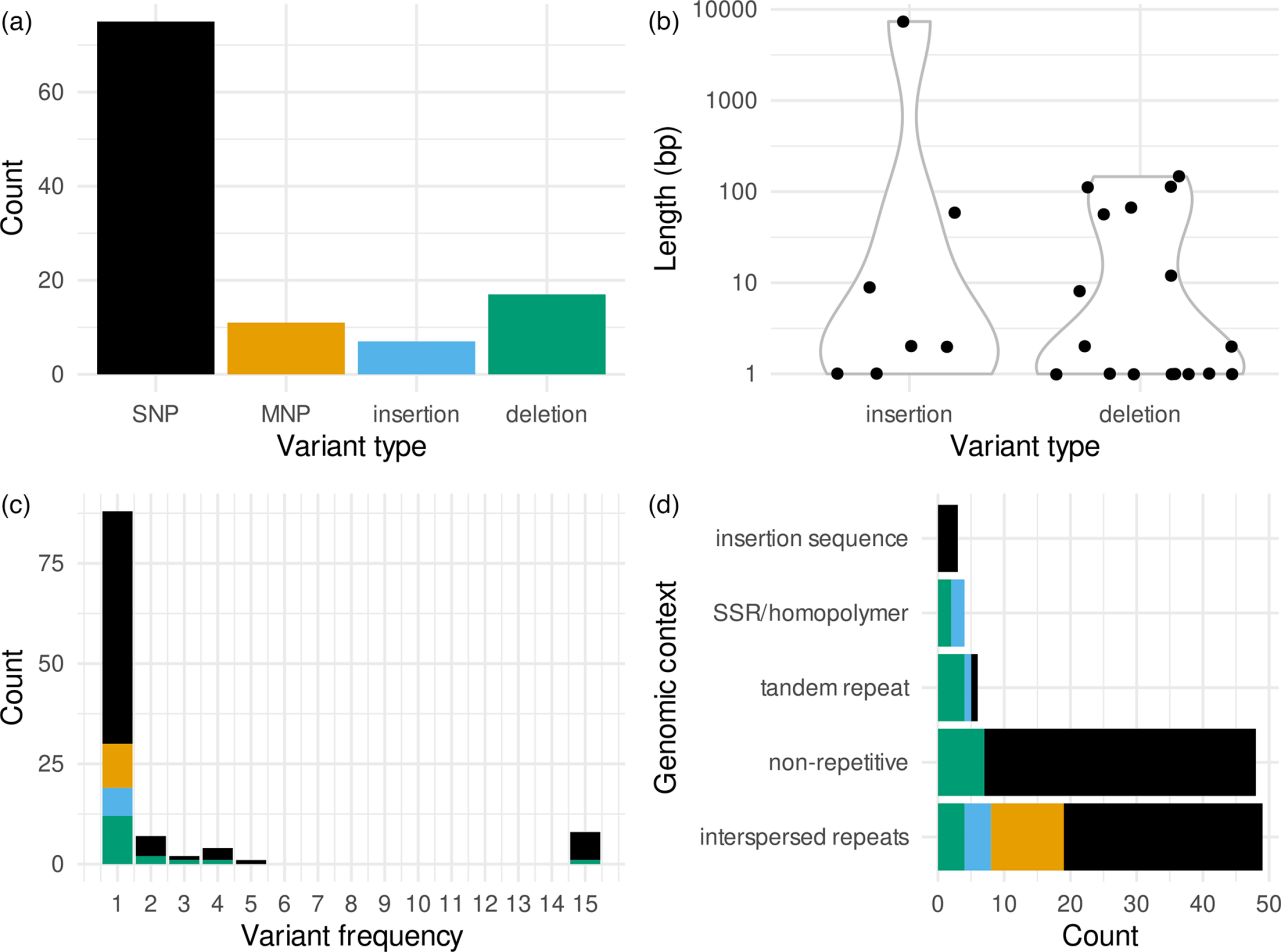
Types of DNA polymorphisms in the 16 sampled strains. (**a**) Counts of SNPs, MNPs, insertions and deletions. (**b**) Length of insertions and deletions, with a log scale on the y-axis. (**c**) Frequency of the variants in the 16 strains. (**d**) Intersection of the variant sites with the annotated repeats.

After having established that the assemblies are essentially error-free and can be trusted, and equipped with a basic understanding of what types of repeats are located where in the genome, we constructed a pangenome graph from the 16 assemblies and identified ‘bubbles’ in the graph (that is, graph regions with alternative paths that represent genetic variation). One hundred ten variants at 109 sites were identified (Fig. 2a, Table S2), including 75 SNPs, 11 multinucleotide polymorphisms (MNPs; simultaneous changes of 2 or 3 bp), 17 deletions and 7 insertions. No insertion sequence polymorphisms or inversions were observed in our sample. Deletion and insertion lengths range from 1 to 7,346 bp, the majority being indels smaller than 10 bp (Fig. 2b). Regarding the frequency of the variants, 88 of 110 (80%) are singletons; that is, they are present in only one of the sampled strains, 14 are shared between up to five strains and eight are present in all but P034-N1426, the strain that diverged early from the rest of the sample (Figs 2c and S1).

One surprising pattern that we observed was an accumulation of seven different missense mutations in grcC1 (Rv0562), five of them singletons and two shared between two strains (Table S2). A closer look at the variants showed that four of them occur at intermediate frequencies within isolates (Fig. S4). Three independent mutations changed the phenylalanine at position 145, suggesting convergent selection on grcC1. Surprisingly, whilst these variants are well supported by the long reads, only two of them were also present in the short reads (Fig. S4). Adaptation to culture media could explain this discrepancy as well as the intermediate frequencies of several variants. We did not follow up this hypothesis in the present study but mention it here because the shared variants in grcC1 do affect the phylogenetic tree estimated in this low-diversity context.

To identify repeat-associated mutations, we intersected the variant sites with our repeat annotation. Sixty-two of the 110 (56%) variants are associated with repeats, whilst 48 occur in non-repetitive genic or intergenic regions (Fig. 2d). For most repeat-associated variants, the annotation directly suggests an underlying mechanism: of the seven insertions and deletions larger than 50 bp, five locate to TRs, with the length of the variants corresponding to a multiple of the repeat periodicity. Four small indels are located in SSRs and homopolymers, suggesting strand slippage as the underlying mechanism. The most striking pattern, however, is the large number of variants that intersect with interspersed repeats. Forty-nine variants (45%), including all MNPs, locate to interspersed repeats. This is a more than 20-fold over-representation, considering that these regions make up 2.1% of the genome.

### Gene conversion between PE/PPE genes accounts for more than a third of all variants

**Fig. 3.**
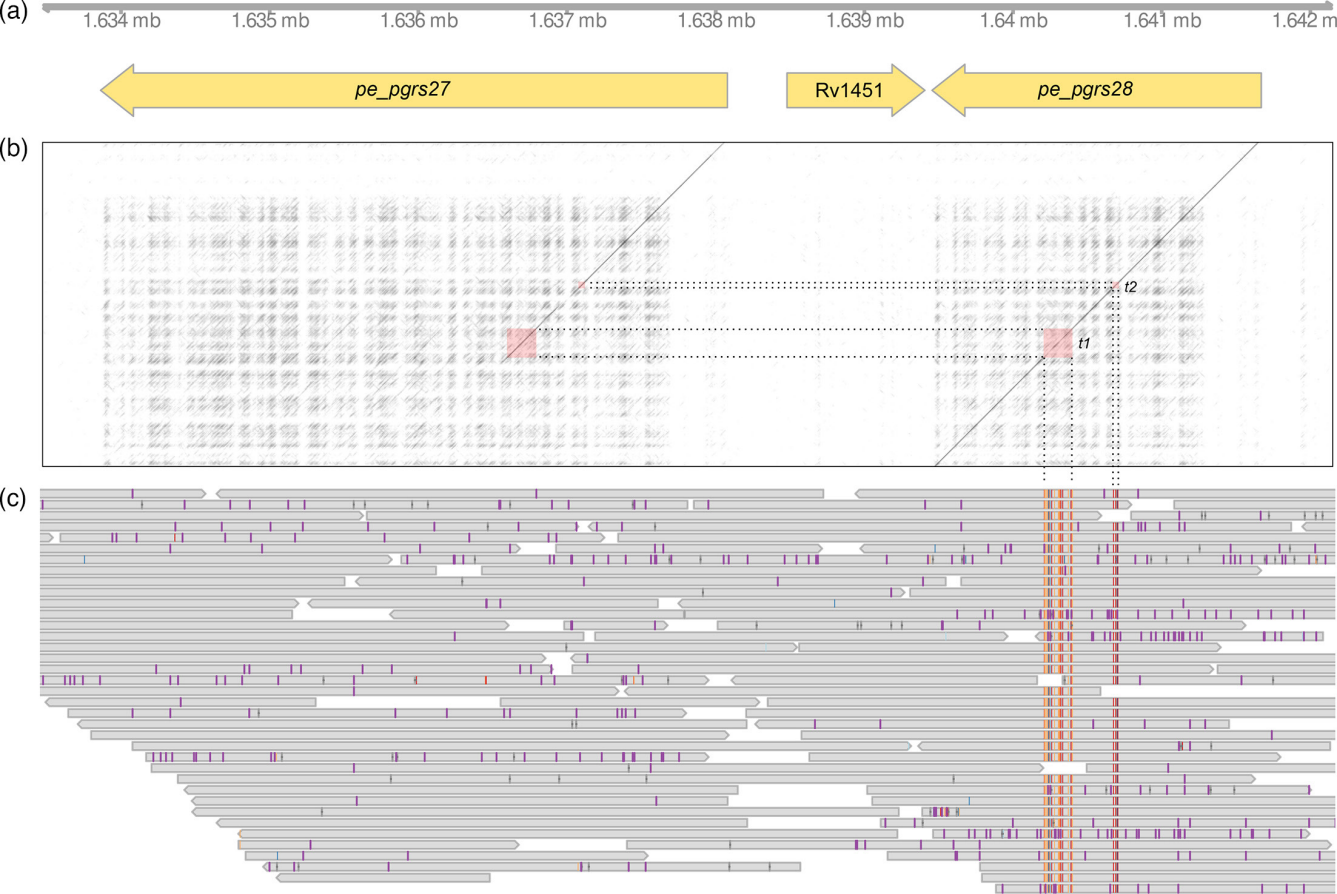
Gene conversion event between *pe_pgrs27* and *pe_pgrs28* in the strain P059-N1392. (**a**) Genes annotated in the affected region. (**b**) A sequence dot plot of the whole region in P059-N1392 on the x-axis and the sequence of the unconverted target gene *pe_pgrs28* on the y-axis. Note the highly repetitive PGRS domains in the C-terminal regions of the two genes. The red squares show the two conversion tracts, t1 and t2, which were copied from the PGRS domain of *pe_pgrs27* to the PGRS domain of *pe_pgrs28*. Both t1 and t2 yield exact blast hits in *pe_pgrs27*. (**c**) PacBio reads of P059-N1392 (gene conversion) aligned against P034-N1426 (ancestral state), highlighting the 34 variants in the two conversion tracts.

A closer look at the variants occurring in interspersed repeats shows that they occur as dense clusters of variants in single strains and are located in PE/PPE genes, two multigene families that are characteristic of pathogenic mycobacteria and play a role in host-pathogen interactions. Thirty-two of the variants identified in P059-N1392 cluster in the repetitive C-terminal domain of *pe_pgrs28* (Fig. 3). Similarly, six variants in P003-N1374 cluster in *ppe18*, and four variants cluster in P052-N1429 in *ppe19*. Since *ppe18* was annotated as a surface antigen, we further investigated the variants in this gene and found that two non-synonymous mutations affect two distinct epitope regions (Fig. S5).

Given that these variant clusters occur in interspersed repeats, we hypothesized that they were caused by gene conversion between close paralogs. To test this, we blasted the suspected conversion tracts against the genome, expecting two exact hits in the two paralogs involved versus only a single exact hit in the source gene in strains where no gene conversion occurred. Indeed, the conversion tract in *pe_pgrs28* yields two exact matches in *pe_pgrs27* (Fig. 3). The matching regions are separated by 286 bp, suggesting that the conversion tract is not continuous but interrupted by a stretch of the target gene. The suspected conversion tracts in *ppe18* and *ppe19* yield exact matches in *ppe19* and *ppe18*, respectively – gene conversion has worked both ways in different strains between these two genes, which are 190 kb apart.

### Birth of a new PPE gene through homologous recombination

As noted above, for one strain (P001-N1377), the Flye assembly step resulted in a single non-circularized contig. To understand why the assembly failed in this strain, we aligned the reads of P001-N1377 against a close relative where the non-assembled region posed no problems. Double coverage and clipped reads, where the clipped parts feed back into the repeat on the opposite site, suggest that this region is duplicated in P001-N1377 (Fig. S6). A comparatively long read would be required to resolve this 2×7, 346=14,692 bp region, given an N50 read length of 6,839 bp for this strain. Indeed, there is one read of 18,797 bp that spans the region (Fig. S6). The short overlap on the 3′ side (140 bp) might explain why Flye failed to close the gap.

**Fig. 4.**
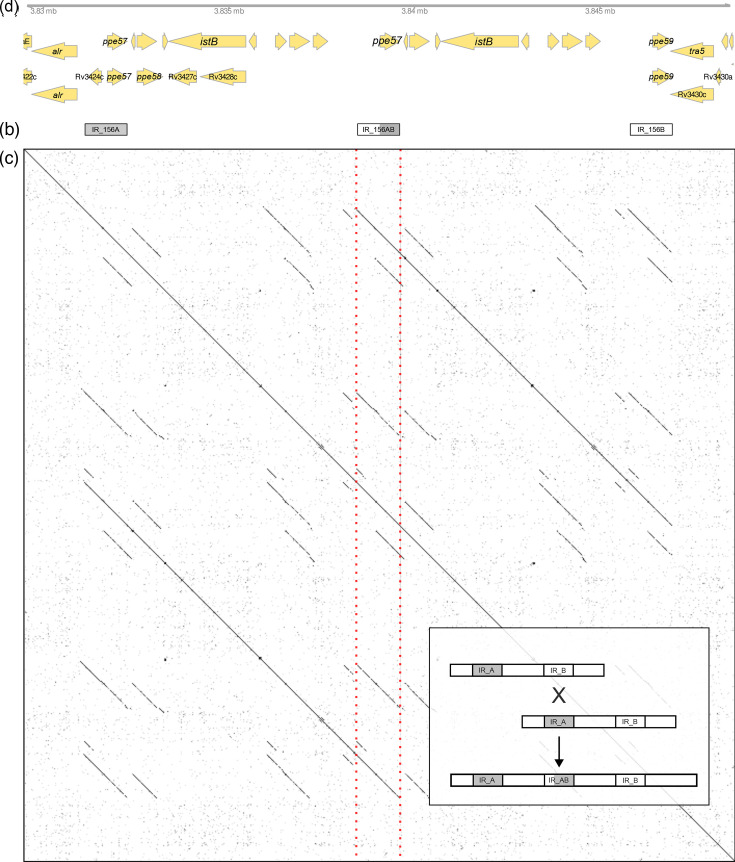
The duplicated region in P001-N1377. (**a**) Genes annotated in the duplicated region, above our *de novo* annotation, below a liftover from the H37Rv reference annotation. Note that H37Rv genes were lifted over only once, such that the duplicated region remains unannotated. (**b**) Interspersed repeats annotated in this region. (**c**) Sequence dot plot of the duplicated region against itself. The red vertical lines highlight the centre of the duplication where the duplicates overlap and the recombinant interspersed repeat is located. (**d**) A model for the origin of the observed duplication through homologous recombination. Note that the interspersed repeats include two PPE genes but are not equal to the gene boundaries. The new PPE gene is identical to *ppe57* but has the 5′ context of *ppe59*.

The duplication occurred in a region of the genome that contains multiple PE/PPE genes and insertion sequences. According to our *de novo* annotation, the duplication contains nine coding sequences (CDS) (Fig. 4a): *ppe57* (+ strand)*,* a short hypothetical protein (-), two CDS homologous to *ppe58* (+), two CDS that are part of an IS21 insertion sequence (-), a PE-domain containing gene (+), and two PPE genes (+). The latter three CDS are not annotated in H37Rv; their sequences are similar to the closely related *ppe57*, *ppe58* and *ppe59* (Fig. 4c), suggesting that they are leftovers from previous duplication events.

The annotated interspersed repeats in the duplicated region proved informative when trying to understand how the duplication might have originated. An interspersed repeat is present in the region, IR-156A and IR-156B (Fig. 4b); the two segments comprise *ppe57* and *ppe59*, respectively, and an additional stretch at the 5′ side of these genes. A blast search of these segments against the graph reveals a third segment at the very centre of the duplication, highlighted by the red lines in Fig. 4(c), which is a recombinant between the two parental segments (Fig. 4a). The location of the recombination breakpoint is unclear but has to be located before or within the first 70 bp of the new, identical copy of *ppe57*, as *ppe57* and *ppe59* are identical for the first 70 bp but then differ substantially. The presence of a third homology segment at the centre of the duplication suggests that the duplication has originated through homologous recombination (Fig. 4d).

### Genetic distances and tree resolution with complete assemblies

**Fig. 5.**
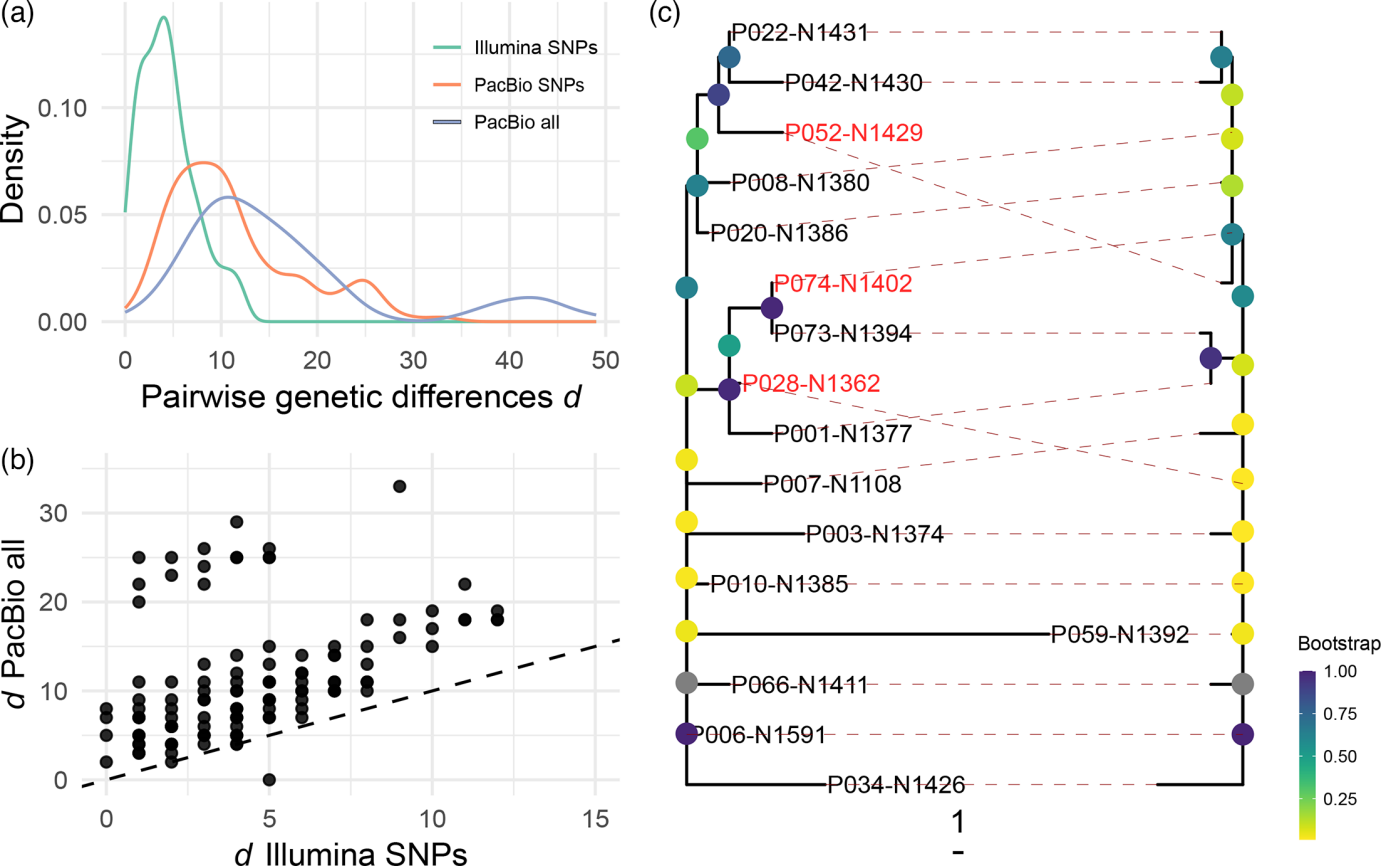
Genetic distances and phylogenetic resolution with complete genomes. (**a**) Distributions of pairwise genetic distances calculated from SNPs in non-repetitive regions (‘Illumina SNPs’), SNPs in all regions (‘PacBio SNPs’) and all variant types in all regions (‘PacBio all’). (**b**) All variants in any region (y-axis) compared to SNPs in non-repetitive regions (x-axis). The dashed line shows unity (intercept 0, slope 1). (**c**) Bootstrapped neighbour-joining trees for all variants (left) and Illumina SNPs (right). Isolates with a different position in the tree are shown in red.

Additional genetic information is valuable in bacterial pathogens like the MTBC, where extreme similarity and lack of temporal signal make it difficult to infer short-scale evolutionary dynamics. As a final analysis, we investigated to what degree long-read sequencing increases genetic resolution. We compared genetic distances *d* based on Illumina reads [13] to distances derived from the pangenome graph. The former is SNP distances in non-repetitive regions, whilst for the latter, we calculated, across the whole genome, both SNP distances (‘PacBio SNPs’) and overall distances that include any variant type (‘PacBio all’, see the Methods section). Fig. 5 shows the expected result that pairwise distances are larger when considering complete genomes: median *d* more than doubles from 4 to 10 when considering SNPs in any region and further increases to 13 when also considering indels and structural variants. The PacBio distance distributions are bimodal: the second modes are comparisons with P059-N1392, the strain in which *pe_pgrs28* was affected by gene conversion (Fig. 3). Gene conversion can introduce multiple variants at once and may thus strongly inflate *d*, as apparent in the cloud of points with disproportionally high PacBio distances in Fig. 5(b). For one single comparison, P073-N1394 vs. P074-N1402, Illumina SNP distances (5) are larger than PacBio distances (0; Fig. 5b). As shown by the phylogenies described in the next paragraph, P074-N1402 is probably not the same isolate in the two studies.

We next estimated neighbour-joining trees in order to find out to what degree the additional variants improve tree topology. Seven missense mutations in the gene *grcC1* were excluded for this purpose as they showed evidence for convergent selection and could confound ancestral relationships (Fig. S4). The tanglegram in Fig. 5(c) shows the tree based on any variant in any region on the left and the tree based on Illumina SNPs on the right. As expected from the prevalence of singleton variants (Fig. 2d), the main difference between the trees is the longer terminal branches in the tree based on all variants (Fig. 5c). The total tree length more than triples from 29 to 103, but the internal branches contribute only seven units to this increase. Resolution does increase marginally: two isolates could be placed more precisely in the tree due to (a) two linked variants in a copy of IS6110 (P052-N1429) and (b) a deletion in a homopolymer and three genic SNPs that were not present in the Illumina SNP alignment. In summary, whilst adding repetitive regions and additional variant types led to a considerable increase in genetic distances, it only marginally improved phylogenetic resolution because the vast majority of variants are singletons.

## Discussion

The promise of third-generation sequencing technologies is to resolve repeats and thus to permit a complete picture of genetic variation. In this study, we show that virtually base-perfect MTBC genomes can be assembled from PacBio HiFi reads. Using a pangenome graph framework, we found that regions and variant types typically ignored by short-read sequencing studies contribute more than half of the genetic variation observed in 16 outbreak strains. The focus on a set of closely related strains means that this result can hardly be generalized: had we selected different isolates, or considered a different outbreak, the contribution of repeat-associated mutational mechanisms might look less (or more) impressive. At the same time, the small number of variants and the absence of complex nested or recurring mutations made it possible to investigate the variants at a level of detail that would not have been possible with more diverse isolates. As discussed in the following, these details do matter not only if we are to understand how different mutational mechanisms provide the raw material for the evolution of the MTBC, but also if repetitive regions are to be used in epidemiological and evolutionary inference.

### Recombination re-emerges as an important mechanism in the MTBC

The observation of three gene conversion events, a duplication and several insertions and deletions in TRs in a sample of closely related strains suggests that homologous recombination operates frequently. Whilst point mutations remained the most frequent events (Fig. 2a), recombination affected large regions and contributed disproportionately to genetic variation: three gene conversion events between members of the PE/PPE families (*pe_pgrs27*/*28*, *ppe19* and *ppe18*) account for 40% of the variants identified, whilst the largest variant is a duplication of 7.3 kb that arose from recombination between interspersed repeats encompassing *ppe57* and *ppe59*.

Evidence for gene conversion and homologous recombination was presented in a series of studies in the early 2000s, before the topic largely disappeared with the advent of short-read sequencing. Karboul *et al*. [40] first discussed gene conversion as a potentially important mechanism in the MTBC and described recurrent conversion between the adjacent *pe_pgrs17* and *pe_pgrs18*. Evidence for gene conversion between members of the PPE [41] and ESX [42] families followed, as well as for *pe_pgrs27* and *pe_pgrs28* [43], the gene most strongly affected by gene conversion in our study, and *ppe18* and *ppe60* [44]. With short-read sequencing, the study of repeats and homologous recombination was marginalized. Several studies still investigated ‘recombination’ but used this general term to denote HGT rather than intrachromosomal recombination (e.g. [45,46]). The apparent absence of HGT in the MTBC is expressed in the paradigm ‘TB does not recombine’. As repeats are now coming into focus again, it is important to recall that homologous recombination is a many-sided fundamental mechanism involved not only in HGT, but also in replication and repair, and its underlying pathways are present in the MTBC [47].

Some expectations regarding the occurrence of gene conversion can be formed by considering the distribution of repeats in the genome (Fig. 1). The rate of recombination, and thus gene conversion, decreases exponentially as sequences diverge [48]. Indeed, to our knowledge, all examples of gene conversion in the MTBC involve closely related genes. Even in the large PE and PPE gene families, with 99 and 68 members in the reference strain H37Rv, close homology is restricted to a few pairs and triplets of genes, and to some larger repeats within single genes (*pe_pgrs24*; Fig. S3B). These numbers would be somewhat higher with more permissive thresholds for homology search (e.g. *ppe58* would appear as a paralog of *ppe57* and *ppe59* [49]), but overall, we expect recombination and gene conversion to be restricted to a few hotspot regions.

Whilst the number of genes involved in recombination might be small relative to the size of the PE/PPE gene families, at least some of the variants in these genes will have phenotypic consequences and affect traits of primary interest in MTBC research, including antimicrobial resistance, immune response and virulence. The three main repetitive gene families in the MTBC (PE, PPE and ESX) play important roles in host-pathogen interactions: they code for secretion systems, surface receptors or secreted proteins that interact with the human immune system, and they were instrumental in the evolutionary transition to an obligately pathogenic lifestyle [50]. Even in our small sample of closely related strains, we found two genes affected by gene conversion and duplication, *ppe18* and *ppe57*, for which there is experimental evidence for a role in immune subversion [51,52]. Gene conversion is a cause of antigenic variation in different prokaryote species (reviewed by [53]). Given that both *ppe18* and *ppe57* are part of TB vaccines in active development [54], a better understanding of their molecular evolution would be relevant.

### Making use of the repeatome

After access to dynamic gene families with potential key roles in host-pathogen interactions, increased genetic resolution is a major promise of long-read sequencing [55]. Even when considering SNPs across all non-repetitive regions of the genome, many strains of the Bernese outbreak still appeared identical, and transmission chains remained unclear, leading Stucki *et al*. to emphasize ‘the need to include repetitive regions’ [13]. In our sample, including repetitive regions and variant types other than SNPs marginally improved phylogenetic resolution (Fig. 5). Most of the interesting, repeat-associated genetic variation identified in this study does not improve resolution because it is present in single strains. This may partly reflect our sampling, and more informative variants would have been found in the full outbreak sample. At the same time, allele frequency distributions in the MTBC do show an extreme shift towards rare alleles [10], and for insertions and deletions, this pattern may even be more extreme given that they tend to be more deleterious [56]. At least partly, polytomies in outbreak trees reflect the biology of the MTBC and not lack of data.

There are probably more straightforward ways to increase phylogenetic resolution than by including a small proportion of the genome that evolves in complex ways. Our repeat annotation suggests that the actual repeat content in MTBC genomes is in the order of 2–3% (Fig. 1) rather than the frequently mentioned 10% – a number arrived at by considering all IS and PE/PPE genes repetitive. Recent studies have already corrected this picture by showing that many PE/PPE genes can be bridged with short reads [57,58], as expected from the sequence divergence in these families [49]. Better resolved trees could thus already be obtained by excluding only what is truly repetitive. Furthermore, indels constitute a large, rarely used source of information in short reads. They can be modelled alongside SNPs in a Bayesian framework to infer dated trees [59] and inform the reconstruction of transmission networks [60].

If gene conversion is frequent, as we hypothesize, it needs to be accounted for in epidemiological and evolutionary inference. As impressively shown by the example of *pe_pgrs28* (Fig. 3), gene conversion can introduce multiple variants at once. This inflates branch lengths and pairwise genetic distances and could confound transmission studies that use clustering thresholds (Fig. 5). On longer evolutionary timescales, recurrent gene conversion can homogenize paralogs and generate patterns of synonymous and non-synonymous diversity that are not appropriately modelled through nucleotide substitution models. A consequence is increased rates of false positives when trying to infer positive selection on genes with close paralogs [61]. Past studies have reported positive selection on a large number of diverse PE/PPE genes [62,64]. It will be interesting to revisit the question of how selection affects these gene families, taking into account that variants might have arisen through recombination rather than point mutation.

### Duplications are the new dark matter

The accuracy of CCS reads allowed us to forego many of the methodological complexities of previous approaches to long-read assembly. Noisy long reads necessitate high sequencing depths and hybrid approaches that combine long and short reads and multiple assembly and polishing steps (e.g. [7,65]). An error rate of 99.63% at 130-fold Oxford Nanopore coverage translates to >15,000 errors in a 4.4 Mb MTBC assembly [66], such that any biological signal in an outbreak sample would be swamped by noise. In contrast, we found that a CCS read depth of 16× was sufficient to obtain a closed genome with only one single ambiguous position. The high accuracy of CCS reads comes at the cost of shorter read lengths and thus a limited ability to bridge duplications and amplifications [67]. The proportion of long reads might be increased through improved DNA extraction and library preparation [7]. But even with longer reads, whether an assembly can be closed also depends on the frequency and length of duplications and amplifications.

In the strains analysed in this study, we stumbled upon a duplication of 7,346 bp that was bridged by a single read. We propose that this duplication has originated through homologous recombination between two interspersed repeats that encompass *ppe57* and *ppe59* (Fig. 4). Interestingly, this mechanism could result in recombinant genes and thus create ‘new’ genes or regulatory contexts in a clonal organism. Compared to other duplications that have been described in the MTBC, a duplication of 7,346 bp is not particularly large. Well-described duplications are the 30 kb DU1 and 36 kb DU2 duplications in the Bacillus Calmette-Guérin vaccine clade [68], or the massive duplication of 350 kb that includes the DosR regulon [69] and has appeared repeatedly in lineage 2 and lineage 4 [70]. More recent examples are a 38- to 60-fold amplification of *esxR*/*esxS* and flanking PE/PPE genes in H37Rv mutant strains where the ESX-3 excretion system had been deleted [71] and a 120 kb duplication that evolved twice independently during experimental adaptation to biofilm growth [72]. It remains to be seen whether these latter cases are exceptional and arose because of the unusual selective pressures during the experiments, or whether the frequency of duplications in the MTBC has been underestimated because it has not been possible to investigate structural variants systematically.

## Supplementary material

10.1099/mgen.0.001396 Uncited Supplementary Material 1.

10.1099/mgen.0.001396 Uncited Table S1.

10.1099/mgen.0.001396 Uncited Table S2.

## Abbreviations

CCS: circular consensus sequences
HGT: horizontal gene transfer
IS: insertion sequence
MNPs: multinucleotide polymorphisms
MTBC: *Mycobacterium tuberculosis* complex
PacBio: Pacific Biosciences
PGGB: PanGenome Graph Builder
SSRs: short sequence repeats
TB: tuberculosis
TRs: tandem repeats
