# Supplementary material for: Gene conversion and duplication contribute to genetic variation in an outbreak of Mycobacterium tuberculosis
Source: Microb Genom. 2025 May 1;11(5):001396. doi: 10.1099/mgen.0.001396 (PMC12046097; doi:10.1099/mgen.0.001396)
Supplement: Uncited Supplementary Material 1. [file mgen-11-01396-s001.pdf]

Large contribution of repeats to genetic variation in an outbreak of *Mycobacterium tuberculosis*

Christoph Stritt, Michelle Reitsma, Ana Maria Garcia Marin, Galo Goig, Anna Dötsch, Sonia Borrell, Christian Beisel, Iñaki Comas, Daniela Brites, and Sebastien Gagneux

Supplementary Figures

Figure S1: Rooted phylogeny of the Bernese outbreak
Figure S2: Inconsistencies between reads and assemblies
Figure S3: Characteristics of SSRs, TRs, and interspersed repeats
Figure S4: Evidence for recent convergent selection on *grcC1*
Figure S5: Missense variants in epitope regions of *ppe18*
Figure S6: IGV visualization of the duplication containing *ppe57*

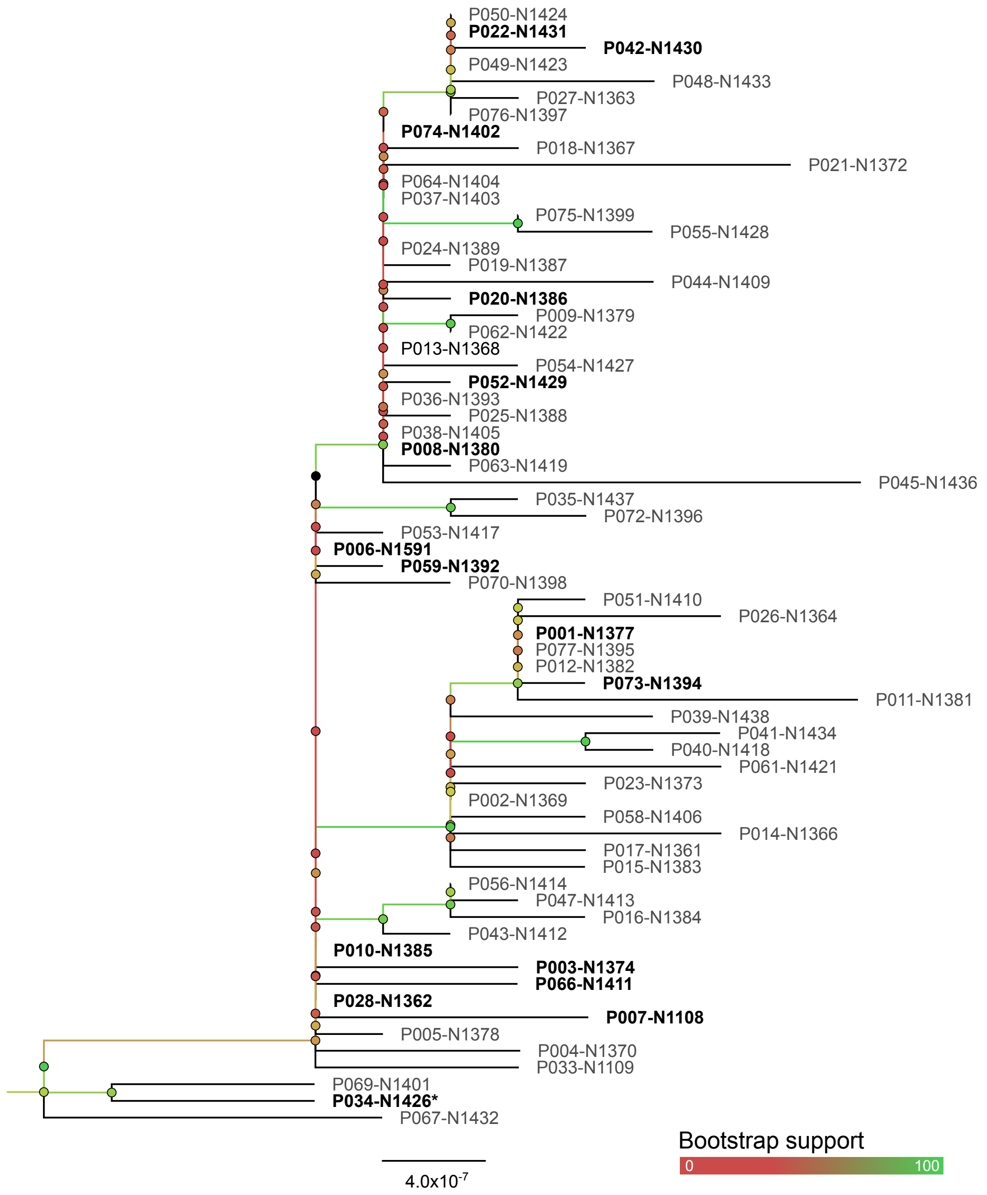

Figure S1: Rooted maximum-likelihood phylogeny of the Bernese outbreak, based on public short reads (PRJEB5925, Stucki et al. 2015). The strains in bold were chosen for PacBio sequencing. 100 bootstrap replicates were performed, internal nodes are colored according to the bootstrap support.

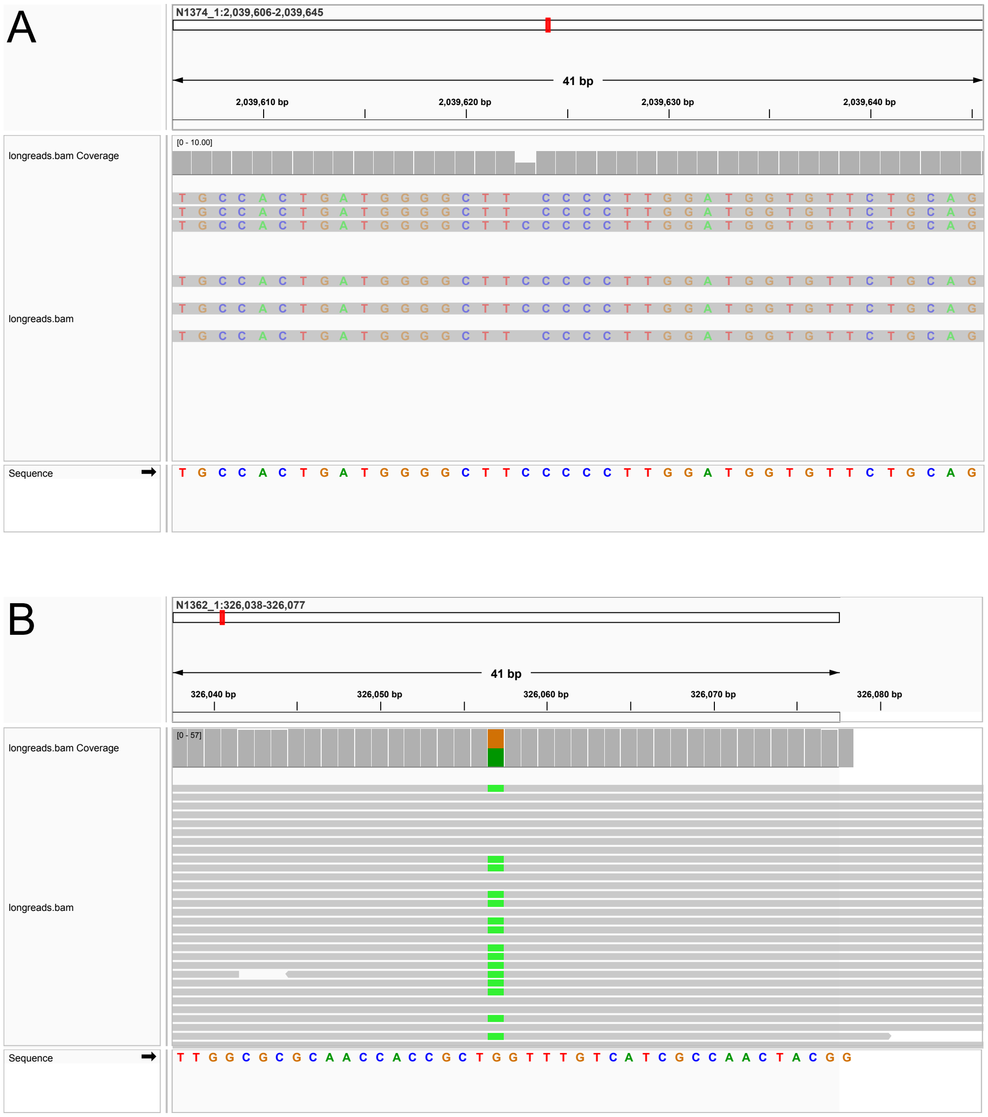

Figure S2: Inconsistencies between long reads and the genomes assemblied from them. Different reasons for the inconsistencies are apparent when aligning the reads to the assemblies and visualizing them with IGV: A) low coverage, B) genuine heterogeneity, C) misassemblied duplication, D) misassembled single-base insertions.

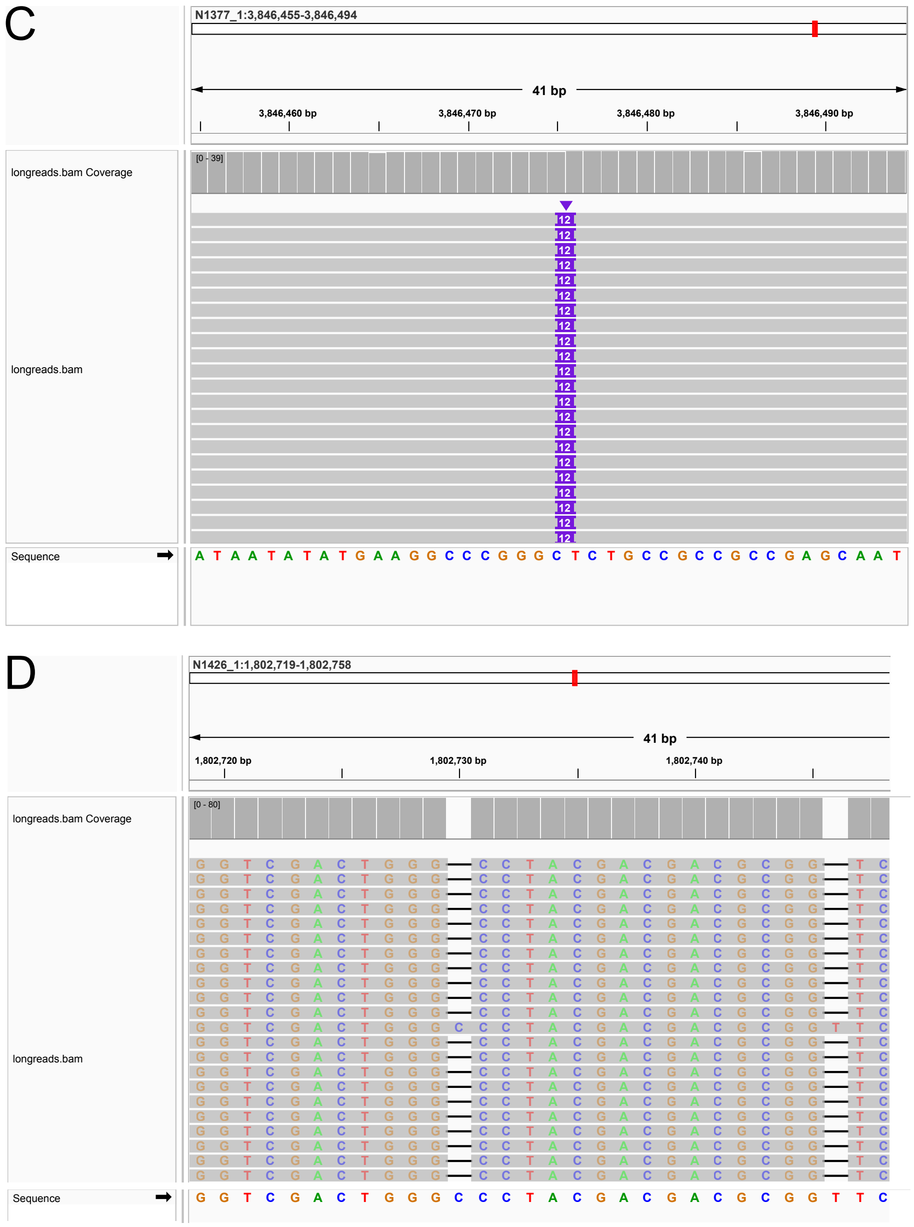

Figure S2: (continued).

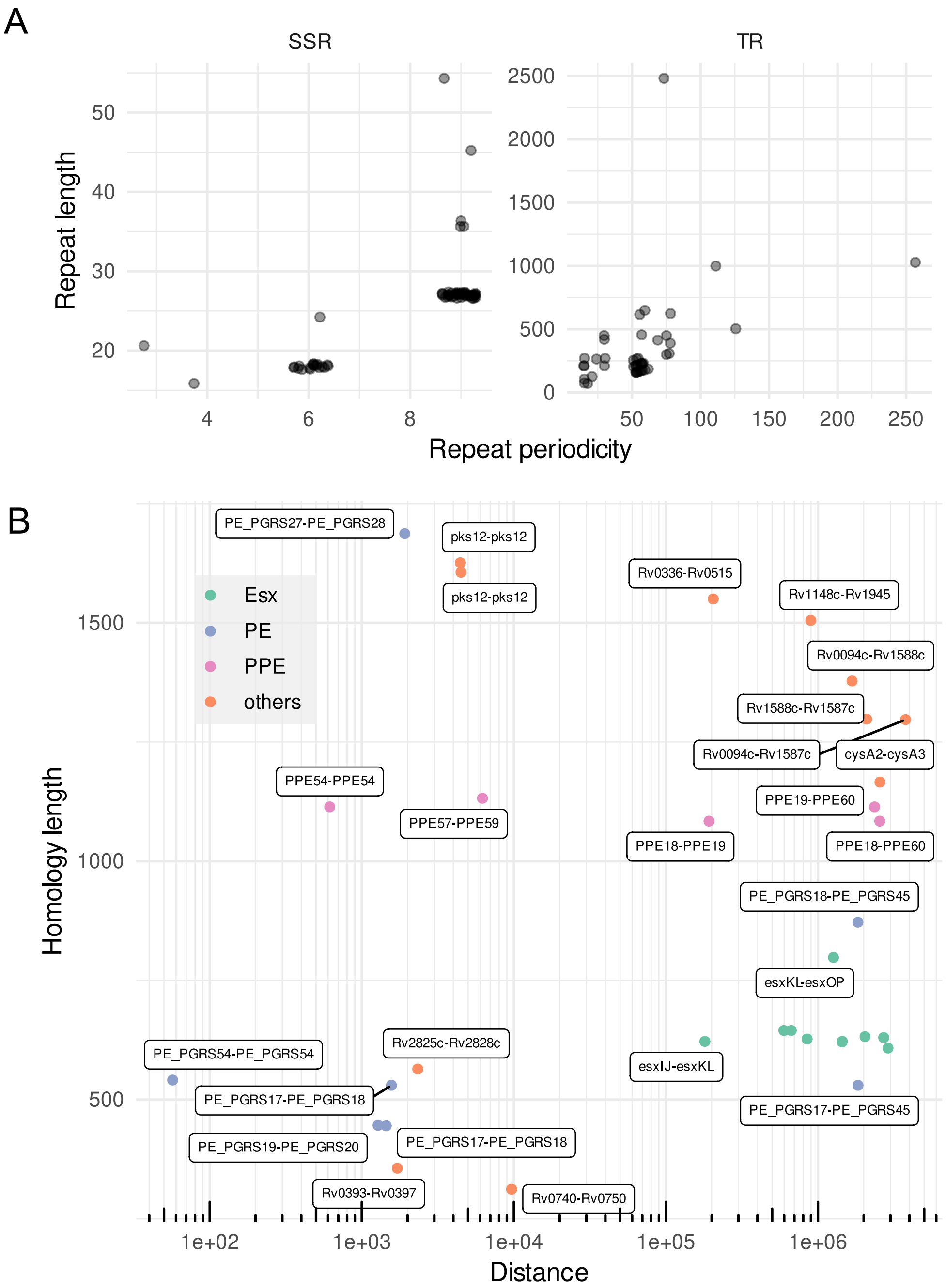

Figure S3: Characteristics of SSRs, TRs, and interspersed repeats. A) Periodicity and total length of short sequence repeats (SSRs) and tandem repeats (TRs). B) Gene pairs sharing substantial homology (over at least 20% of their length). The x-axis shows the distance between the pairs, the y-axis the length of the homology segment. Colors indicate the three main repetitive gene families in the MTBC. Not all esx pairs are labeled due to lack of space. Genes of unknown function are designated by gene names beginning with "Rv".

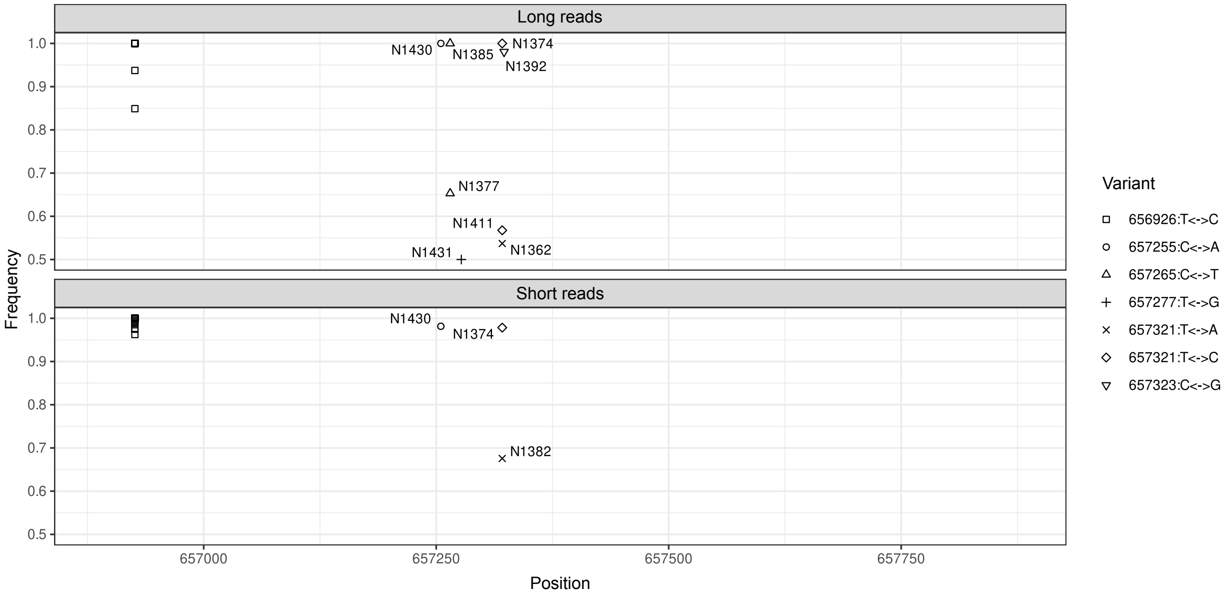

Figure S4: Evidence for recent convergent selection on *grcC1* (Rv0562). Long and short reads were aligned against P034-N1426 with minimap2 v. 2.24-r1122 , and variants were called with freebayes v. 1.3.4. Only genotypes called as '1' are displayed, which corresponds to majority variants that are also present in the assemblies. The x-axis shows the position along the gene, the y-axis the frequency of the variant within the isolates (that is, the proportion of reads supporting the ALT vs. the REF allele). Variants present in the long reads for the 16 strains are shown in the upper panel, variants in the short reads for the 68 strains sequenced by Stucki et al. in the lower panel. The left-most variant, 656926:$T \leftrightarrow C$, is a mutation in the reference and was called in all other strains. Three independent mutations, 657,321:$T \leftrightarrow A$, 657,321:$T \leftrightarrow C$, and 657,323:$C \leftrightarrow G$, change the phenylalanine at position 145, the two former to isoleucine and the latter to leucine. Only two fixed variants present in the long reads were confirmed by the short reads, 657,255:$C \leftrightarrow A$ in N1430, and 657,321:$T \leftrightarrow C$ in N1374.

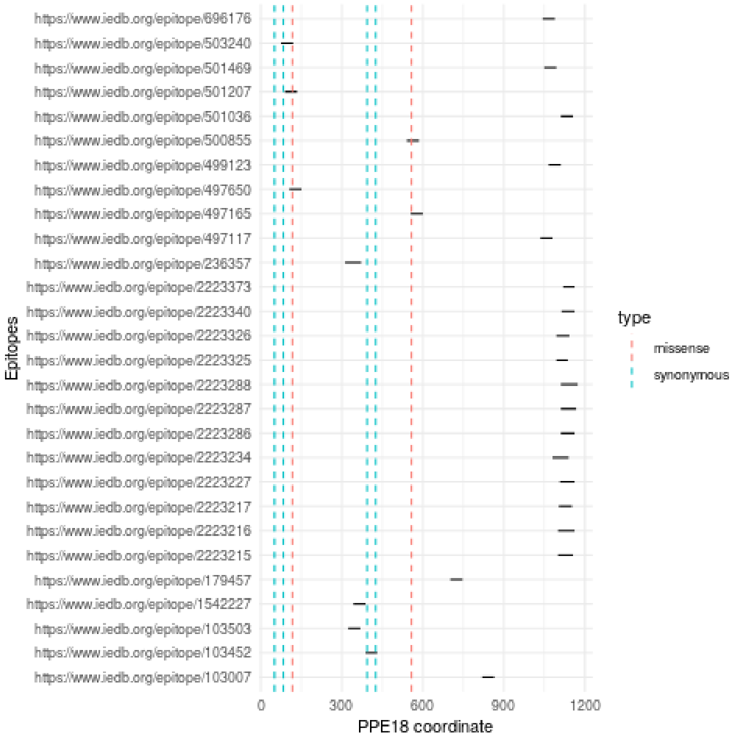

Figure S5: Gene conversion in PPE18 has introduced two missense variants in two epitope regions. The different epitopes downloaded from the IEDB are shown on the y-axis, their position is shown along the x-axis.

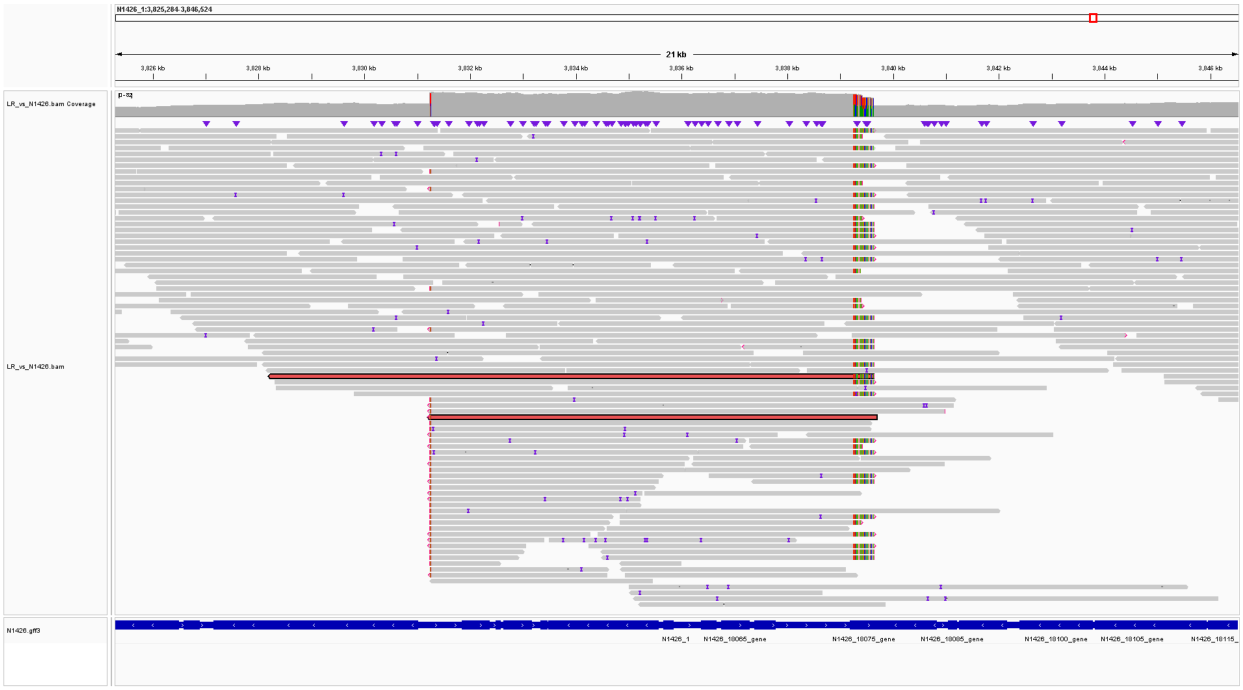

Figure S6: Long-reads from P001-N1377 containing the duplication aligned against the genome of P034-N1426 that does not contain the duplication. The single read of 18,797 bp spanning the whole region is highlighted in red.
